# Genetic Polymorphisms and Kidney Stones Around the Globe: A Systematic Review and Meta-Analysis

**DOI:** 10.3389/fgene.2022.913908

**Published:** 2022-06-30

**Authors:** Abdolreza Mohammadi, Alireza Namazi Shabestari, Leila Zareian Baghdadabad, Fatemeh Khatami, Leonardo Oliveira Reis, Mahin Ahmadi Pishkuhi, Seyed Mohammad Kazem Aghamir

**Affiliations:** ^1^ Urology Research Center, Tehran University of Medical Sciences, Tehran, Iran; ^2^ Department of Geriatric Medicine, School of Medicine, Tehran University of Medical Sciences, Tehran, Iran; ^3^ UroScience and Department of Surgery (Urology), School of Medical Sciences, University of Campinas, Unicamp, and Pontifical Catholic University of Campinas, PUC-Campinas, Campinas, São Paulo, Brazil; ^4^ Pars Advanced and Minimally Invasive Medical Manners Research Center, Pars Hospital, Iran University of Medical Science, Tehran, Iran

**Keywords:** urolithiasis, single nucleotide polymorphisms, genetic polymorphism, recurrent kidney stone, meta-analysis

## Abstract

**Objective:** This study explores associations between recurrent kidney stones and genetic polymorphisms.

**Methods:** Meta-analysis of polymorphisms in renal stone cases versus control groups. Four electronic databases (PubMed, SCOPUS, EMBASE, and Web of Science) were searched up to 30 May 2021, using the keywords: “kidney stone” or “kidney calculi,” or “urolithiasis” or “nephrolithiasis” or “urinary calculi” and “genome” or “genetic” or “mutation” or “single nucleotide polymorphism.” Forrest plots, ORs, 95% CI, Chi-square (χ2)-test, and index of heterogeneity (I2) were calculated. Only studies with Newcastle–Ottawa scale (NOS) ≥ 6 were included for quality control, and Funnel, Begg’s, and Eager’s plots assessed publication bias. PROSPERO: CRD42022250427.

**Results:** Among 7,671 searched articles, 72 were included. Polymorphisms in VDR (OR: 1.20; 95% CI: 1.06–1.36), CASR (OR = 1.24; 95% CI: 1.01–1.52), Osteopontin (OR = 1.38; 95% CI: 1.09–1.74), and Urokinase genes (OR = 1.52; 95% CI: 1.02–2.28) showed a significant association with risk of urinary stone formation, while Klotho gene showed a protective effect (OR = 0.75; 95% CI: 0.57–0.99). The VDR gene polymorphism was frequent in Asians, whereas CASR polymorphism was frequent in European and North American populations.

**Conclusion:** Multifactorial nature of the stone formation, emphasizing the role of environmental factors, might explain contradictory results in the literature. While polymorphisms in VDR, CASR, Osteopontin, and Urokinase genes were associated with urinary stone formation, the Klotho gene showed a protective effect.

## Introduction

Urolithiasis is a worldwide problem with increasing incidence and prevalence (varying worldwide from 5 to 20%) and has substantial health and socioeconomic burden (Trinchieri, 2008; Liu et al., 2018). It is advantageous if, among patients with recurrent kidney stones and their relatives, the clinician could predict patients at high risk that would benefit from tailored therapy, controlled lifestyle, and continual check-ups with necessary organized evidence. Urolithiasis is a complex disease dependent on genetic and environmental factors (Curhan et al., 1993; Eisner and Goldfarb, 2014; Mohammadi et al., 2021).

Single nucleotide polymorphisms (SNPs) are the most common population-dependent genetic variation. SNPs are ethnicity-dependent variations that occur commonly throughout a person’s DNA. They appear almost once in every 1,000 nucleotides, meaning roughly 4 to 5 million SNPs in a person’s genome. In particular, genetic variants in the population may contribute significant risk for manifesting such multifactorial disorders, including kidney stone formation and recurrence (Howles and Thakker, 2020; Singh et al., 2021).

The study of associated SNPs will assist in identifying individuals at risk of kidney stone recurrence, defining the pathophysiological mechanisms, and determining novel targets for drug therapy (Mittal et al., 2008). However, although several genes associated with recurrent kidney stones have been identified, the results have been inconsistent across studies. Some meta-analyses are available that evaluate one or two targeted genes like *Vitamin D Receptor (VDR), calcium-sensing receptor (CASR),* and *Urokinase* (Li et al., 2013; Amar et al., 2020a; Tabaei et al., 2021). A meta-analysis evaluating the associations between all genetic polymorphisms and kidney stones has not been reported.

Therefore, we performed a systematic review and meta-analysis to comprehensively appraise the existing literature for possible associations between genetic polymorphisms and recurrent kidney stones.

## Materials and Methods

### Search Strategies

We conducted a review following the Systematic Reviews and Meta-Analyses (PRISMA) guideline (Page et al., 2021). The review was registered at the International Prospective Register of Systematic Reviews (PROSPERO: CRD42022250427) https://www.crd.york.ac.uk/prospero/display_record.php?RecordID=250427. Four electronic databases, PubMed, SCOPUS, EMBASE, and Web of Science, were used, and the last search update was 30 May 2021. The following subject terms and keywords were applied: “kidney stone” or “kidney calculi” or “urolithiasis” or “nephrolithiasis” or “urinary calculi” and “genome” or “genetic” or “mutation” or “single nucleotide polymorphism.” Detailed information regarding the keywords and search hits is presented in (Supplementary File S1).

### Inclusion and Exclusion Criteria

The outcome of interest comprised associations between gene polymorphisms and recurrent kidney stones. Inclusion criteria: data available to determination of odds ratios (OR), 95% confidence intervals, and associated *p*-value (*p* < 0.05). Exclusion criteria: unrelated titles and abstracts, case reports, qualitative studies, *in vitro* experiments, animal studies, conference or poster abstracts, duplicated data, book chapters, letters, reviews, insufficient population demographic data, and unavailability of detailed data or full text.

### Data Extraction

Two investigators independently performed a quality assessment and data extraction under the supervision of a third investigator. The methodological quality of studies was assessed by Newcastle–Ottawa scale (NOS), and only studies with a quality score of 6 or better were considered in the meta-analysis. For all studies, beyond gene polymorphisms and recurrent kidney stones, the extracted information included: the first author, country, continent, ethnicity, and year of publication. We also collected a sub-population of Caucasians for further analysis.

### Statistical Analysis

Effect estimates for Odds Ratio (OR) and its associated 95% Confidence Interval (CI) were calculated individually for each genetic model (alleles and genotypes) using Hardy-Weinberg Equilibrium (HWE) values. Then, Forest plots were used to show the pooled estimates of ORs, and associated 95% CIs served to determine the degree of association between urolithiasis risk and SNPs. The Chi-square (χ2)-test and index of heterogeneity (I^2^) test consider the possibility of heterogeneity between studies. The random-effect approaches were adopted to compensate for the high values of heterogeneities between studies (I^2^ >50%).

We also performed a sub-group analysis on possible sources of heterogeneity, including continent and specific SNPs. Findings for the subpopulation of Caucasian patients were reported in a separate table to present the distribution of genes and SNPs in this particular subgroup. Finally, we used Funnel, Begg’s, and eager’s plots to assess the possibility of publication bias in the present study data, and qualitative approaches were adopted for inferences.

## Results

**FIGURE 1:**
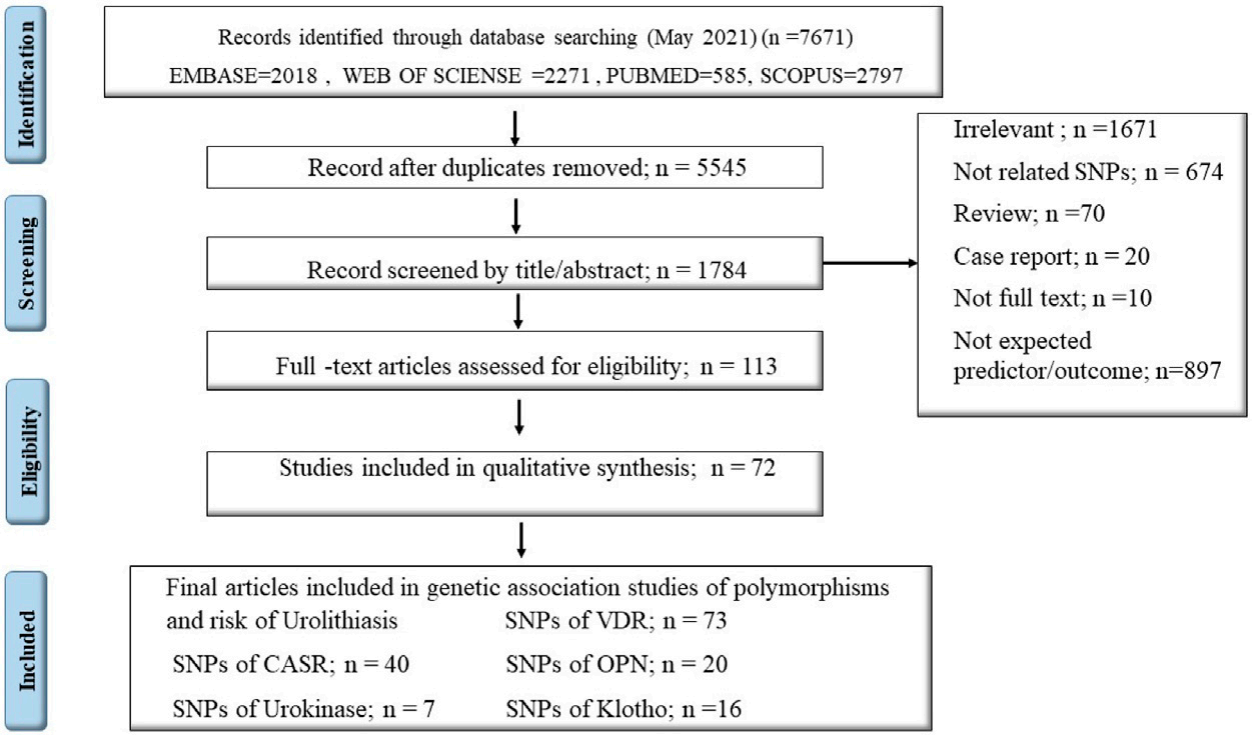
PRISMA flow diagram.

Among a total number of 7671 searched articles in different databases, 156 articles, including *VDR* gene polymorphisms (n = 72), *CASR* (n = 40), *Osteopontin (OPN)* (n = 20), *Urokinase* (n = 7), and *Klotho* gene (n = 16) were eligible for systematic review. Seventy-two articles were included in the meta-analysis to estimate the overall odds ratio (OR) of gene expression in renal stone cases versus control groups (Figure 1). The general estimate of OR for each gene is represented in forest plots, followed by subgroup analysis by continent and SNP subgroups. The observed value of I-square in this meta-analysis was higher than 50%, so the random-effect approach was adopted through analysis. Flowchart one depicts the summary of our essay.

**FIGURE 2:**
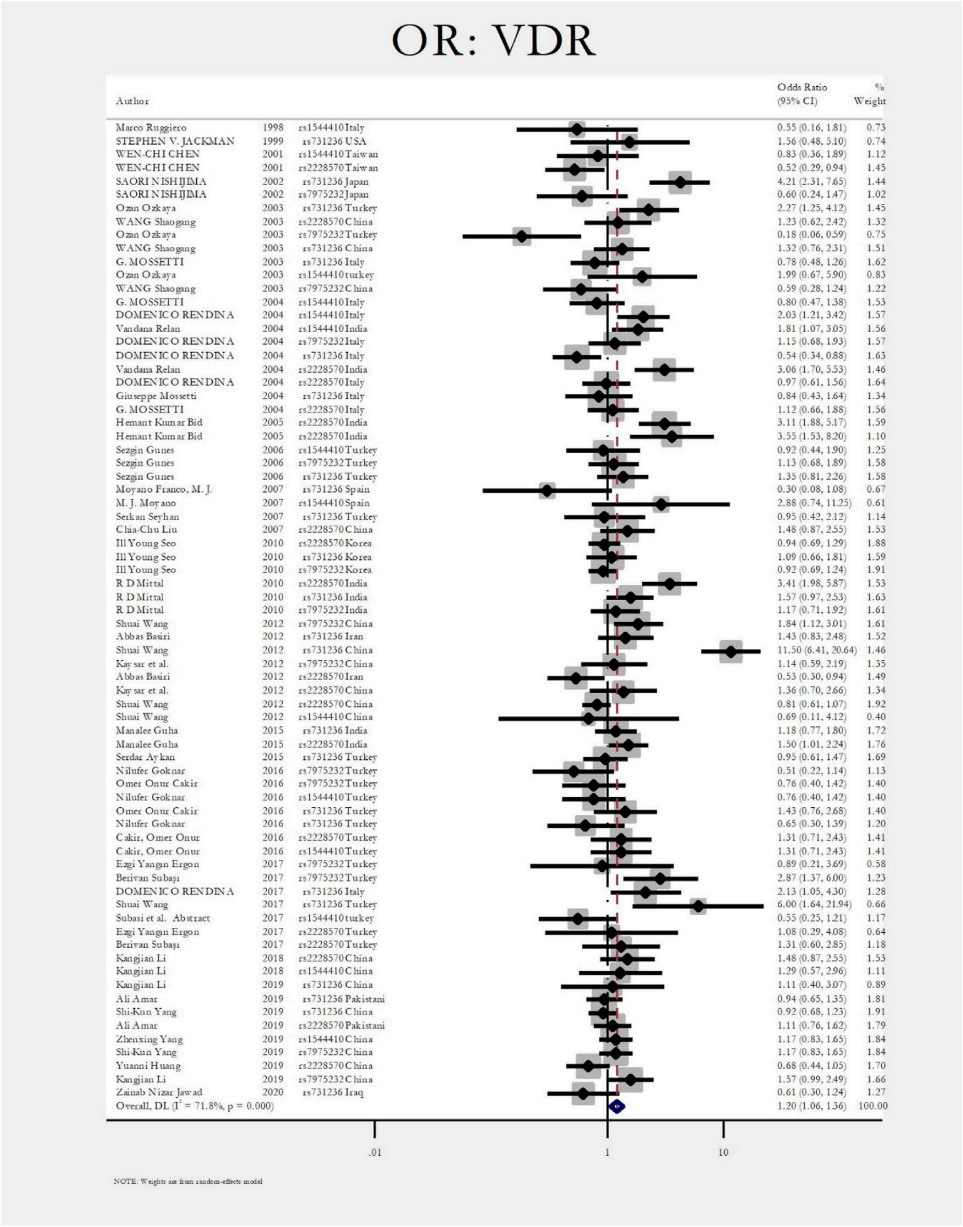
Forest plot: OR for VDR.

**FIGURE 3:**
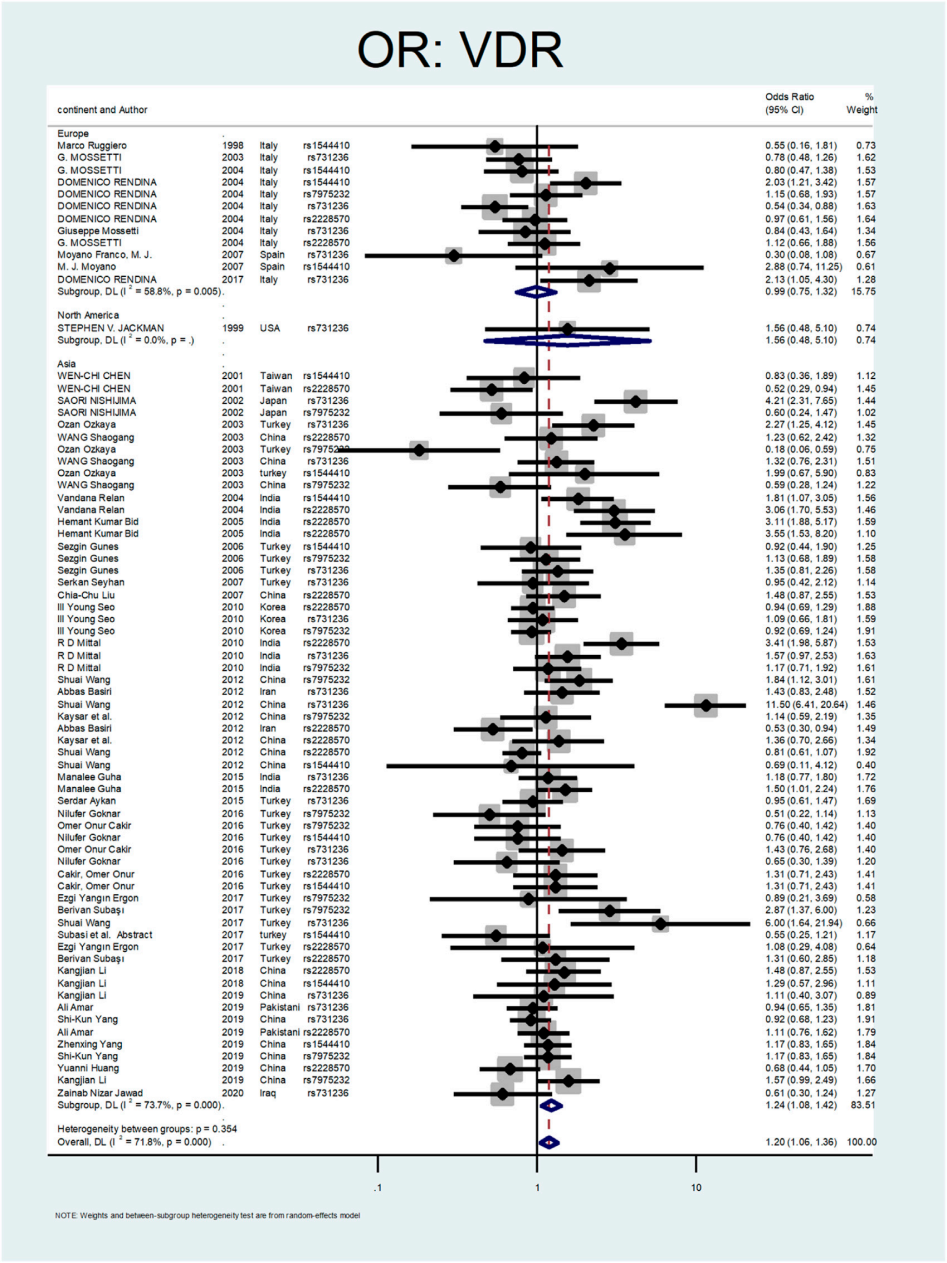
Subgroup analysis for VDR, by continent.

**FIGURE 4:**
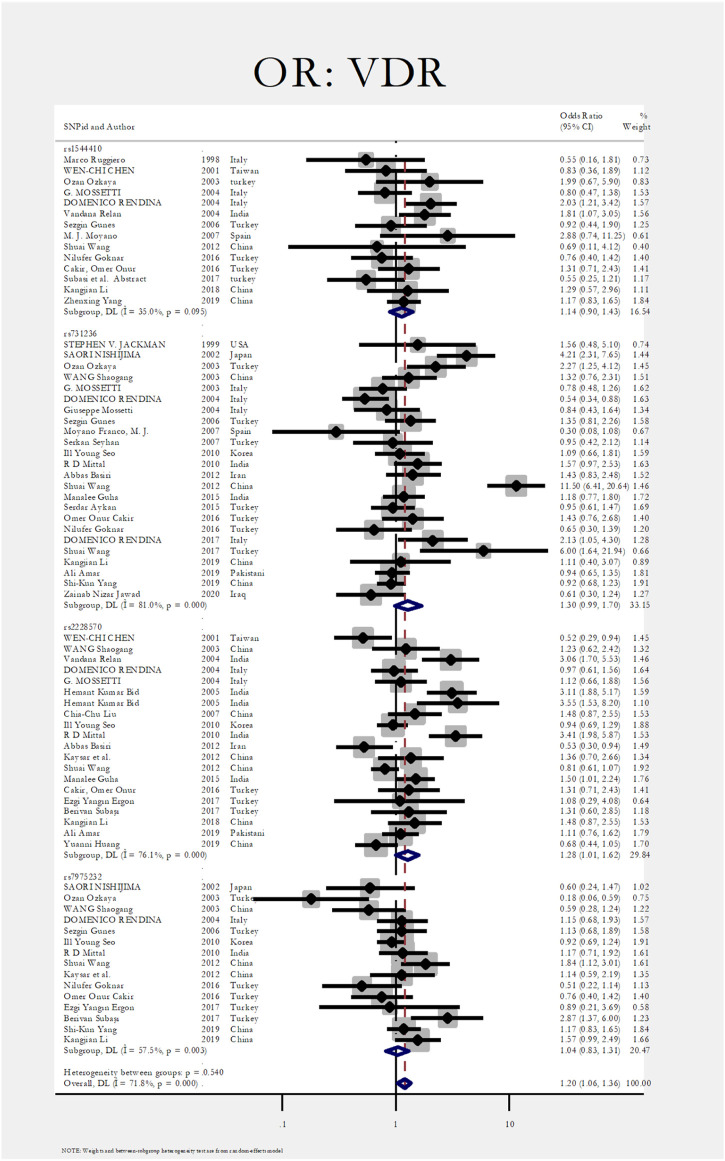
Subgroup analysis for VDR, by SNP subgroup.

Figure 2 shows the pooled estimate of ORs for VDR around 1.20 (95%CI:1.06–1.36), disclosing a significant difference between the two study groups. In Figure 3 and Figure 4, sub-group analysis of this gene showed a significant association in Asian patients with an overall OR of 1.24 (95% CI: 1.08–1.42). However, the estimated OR in European and North American studies subgroups was insignificant (OR = 0.99; 95% CI: 0.75–1.32, and OR = 1.56; 95% CI: 0.48–5.10). Among SNP subgroups, the overall estimate for OR of rs2228570 showed a significant relationship (OR = 1.28; 95% CI: 1.01–1.62). The results for other SNPs, including rs1544410 (OR = 1.14; 95% CI: 0.9–1.43), rs731236 (OR = 1.3; 95% CI: 0.99–1.70, and rs7975232 (OR = 1.04; 95% CI: 0.83–1.31) showed insignificant values.

**FIGURE 5:**
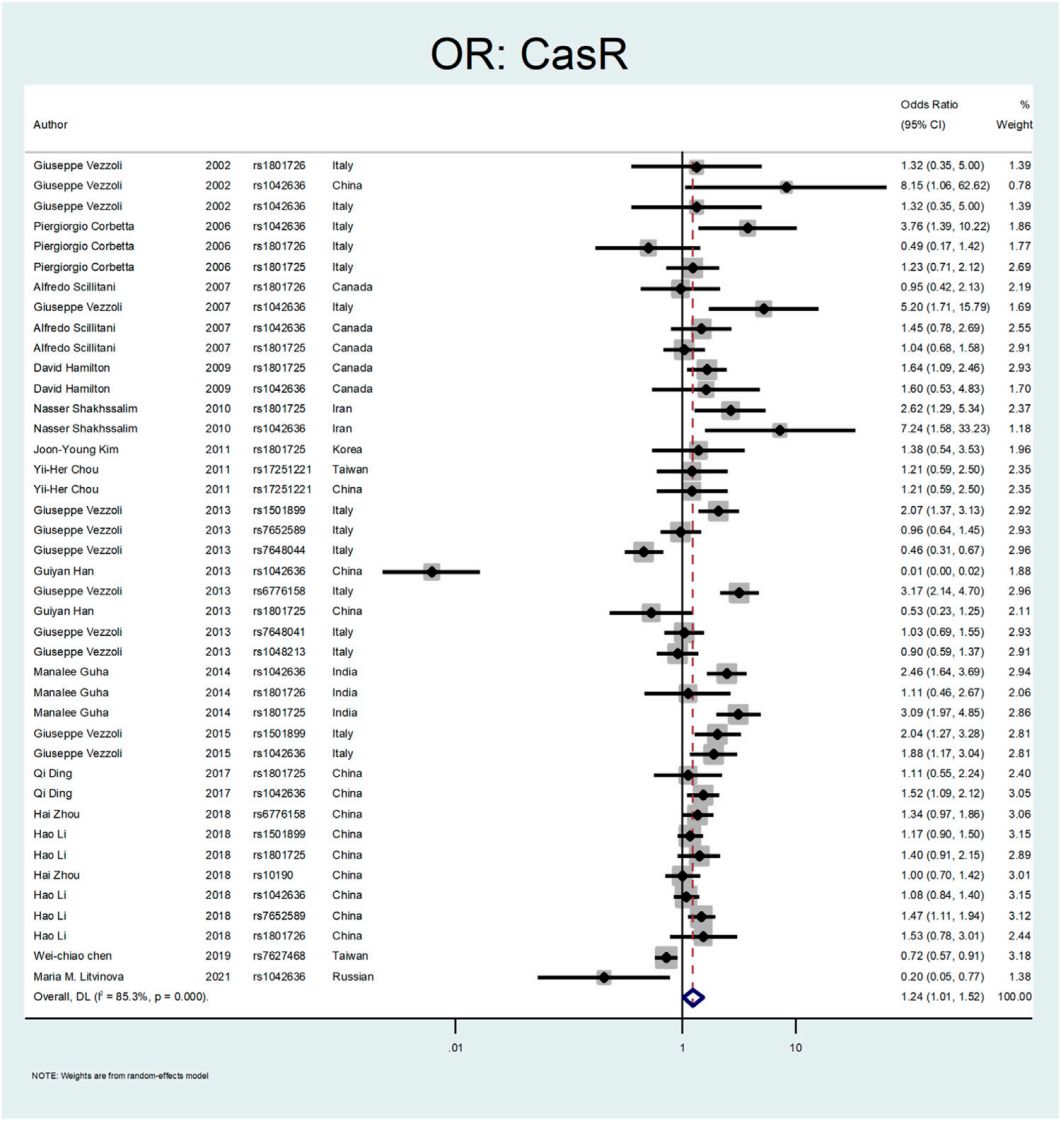
Forest plot: OR for CASR.

**FIGURE 6:**
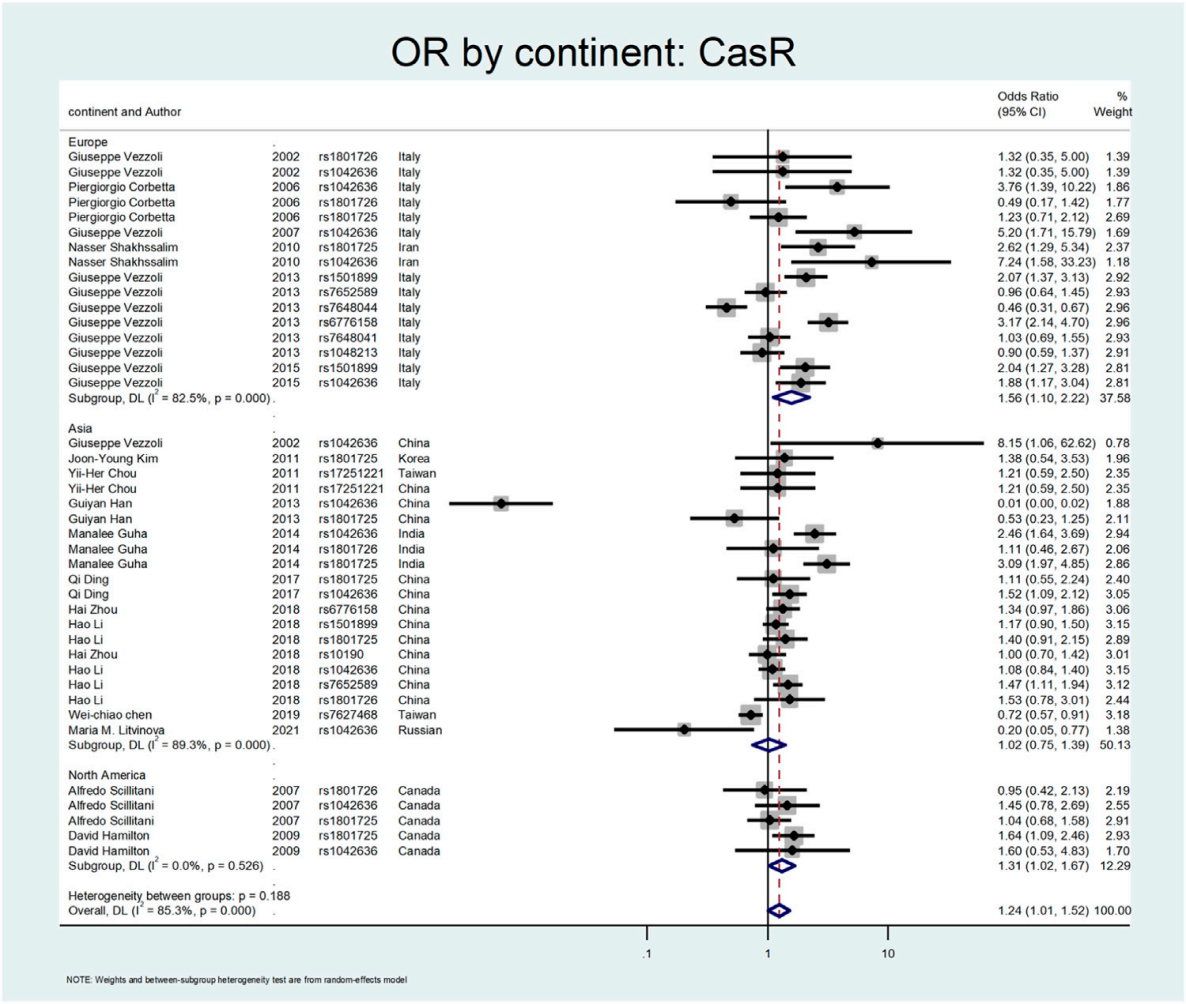
Subgroup analysis for CASR, by continent.

**FIGURE 7:**
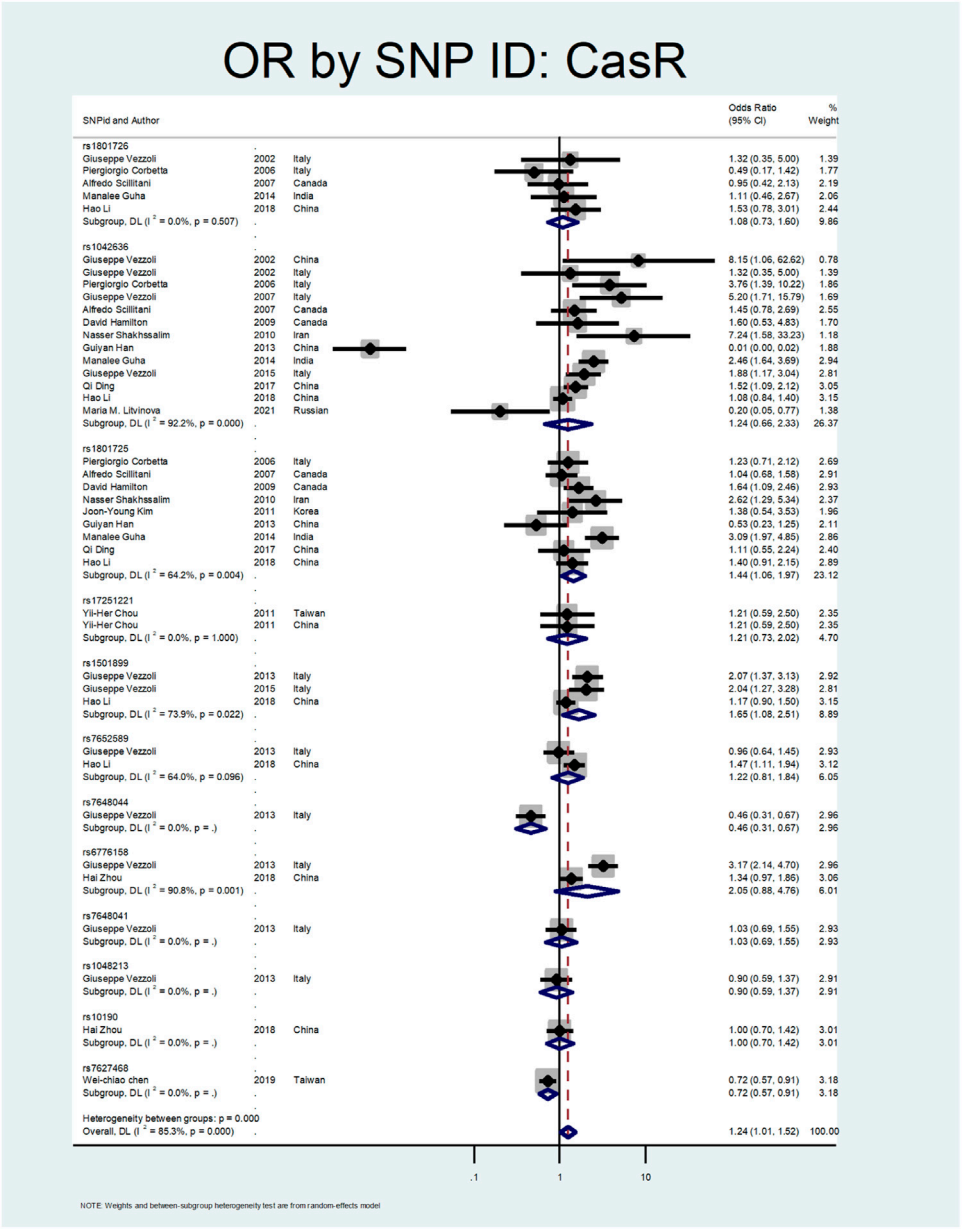
Subgroup analysis for CASR, by SNP subgroup.

Figure 5, Figure 6, and Figure 7 demonstrate the meta-analysis for *CASR*. The overall estimate of OR for this gene showed a significant association (OR = 1.24; 95% CI: 1.01–1.52). The OR in European and North American patients was statistically significant (OR = 1.56; 95% CI: 1.10–2.22, and OR = 1.31; 95% CI:1.02–1.67, respectively), while it was not meaningful in the Asian subgroup (OR = 1.02; 95% CI: 0.75–1.39). With regards to SNP subgroups, the rs1801725 and rs1501899 served as a risk factor with OR values of 1.44 (95% CI: 1.06–1.97) and 1.65 (95% CI: 1.08–2.51), respectively. The rs1042636 and rs1801726 did not show significant odds ratios (OR = 1.24; 95%CI:0.66–2.33, and OR = 1.08; 95% CI: 0.73–1.60, respectively). As well, studies on rs7652589 (OR = 1.22; 95% CI: 0.81–1.84), rs6776158(OR = 2.05; 95% CI: 0.88–4.76), and rs10190 (OR = 1.0; 95% CI: 0.70–1.42) did not show statistically significant association.

**FIGURE 8:**
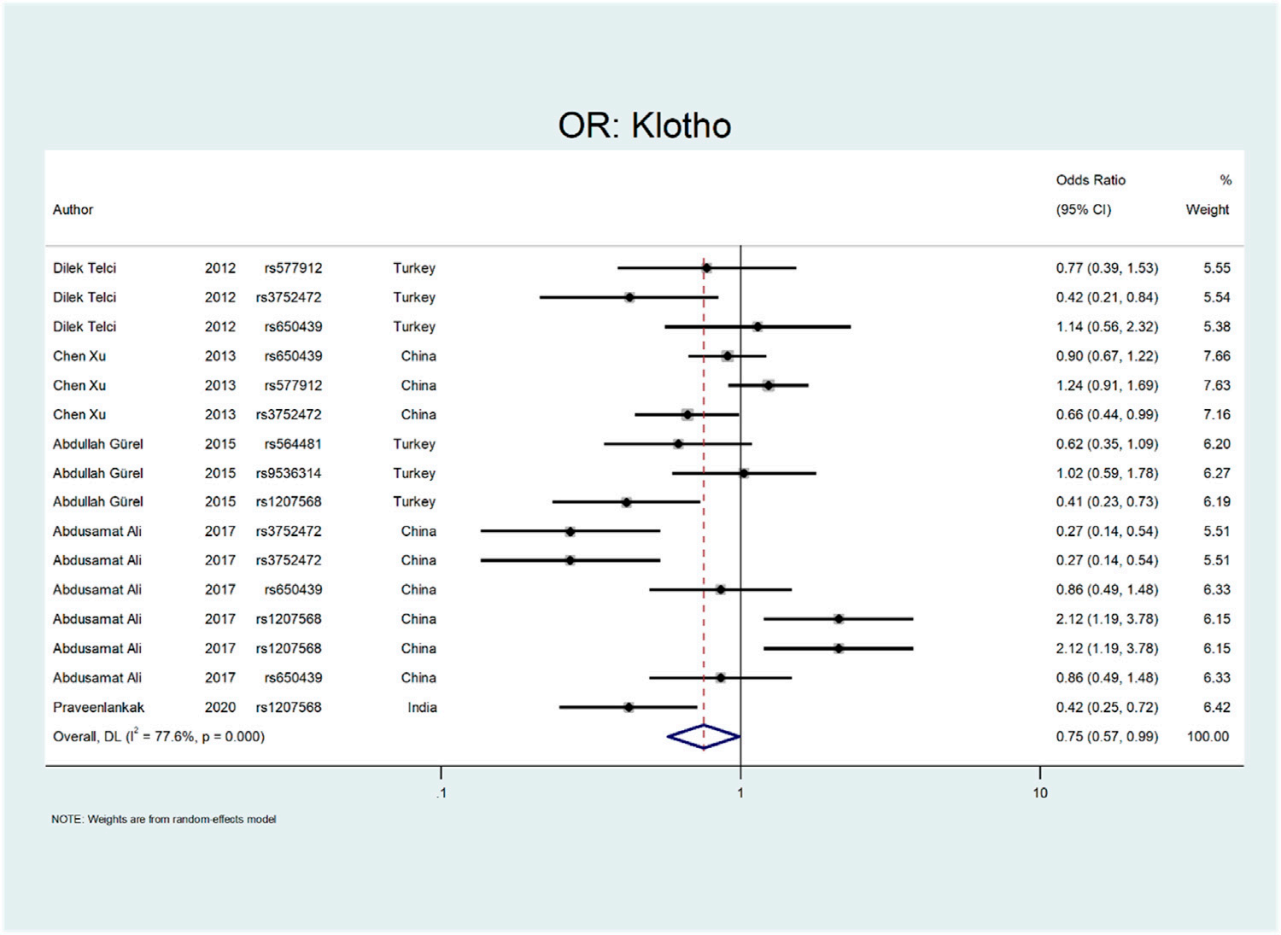
Forest plot: OR for Klotho.

**FIGURE 9:**
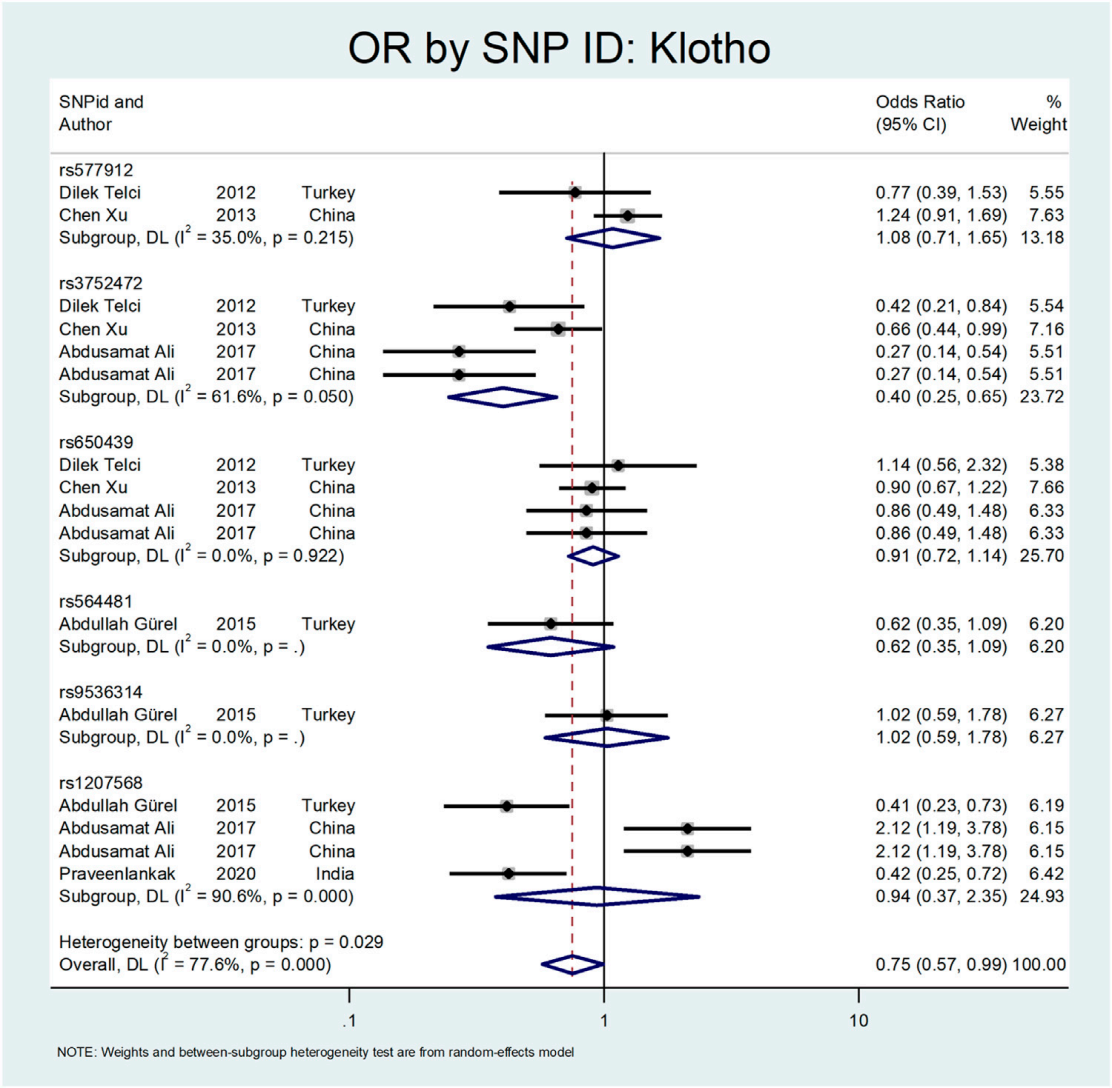
Subgroup analysis for Klotho, by SNP subgroup.

Figure 8 shows the pooled estimate of OR for Klotho gene with protective effect (OR = 0.75; 95% CI: 0.57–0.99). All studies on this gene were conducted in Asian countries, and subgroup analysis by continent was not possible. According to Figure 9, a significant protective odds ratio was observed in rs3752472 (OR = 0.4; 95% CI: 0.25–0.65). Estimated OR for other SNP subgroups including rs1207568 (OR = 0.94; 95% CI: 0.37–2.35), rs650439 (OR = 0.91; 95% CI: 0.72–1.14), rs577912 (OR = 1.08; 95% CI: 0.71–1.65), rs9536314 (OR = 1.02; 95% CI: 0.59–1.78), rs564481 (OR = 0.62; 95% CI: 0.35–1.09) were not significant.

**FIGURE 10:**
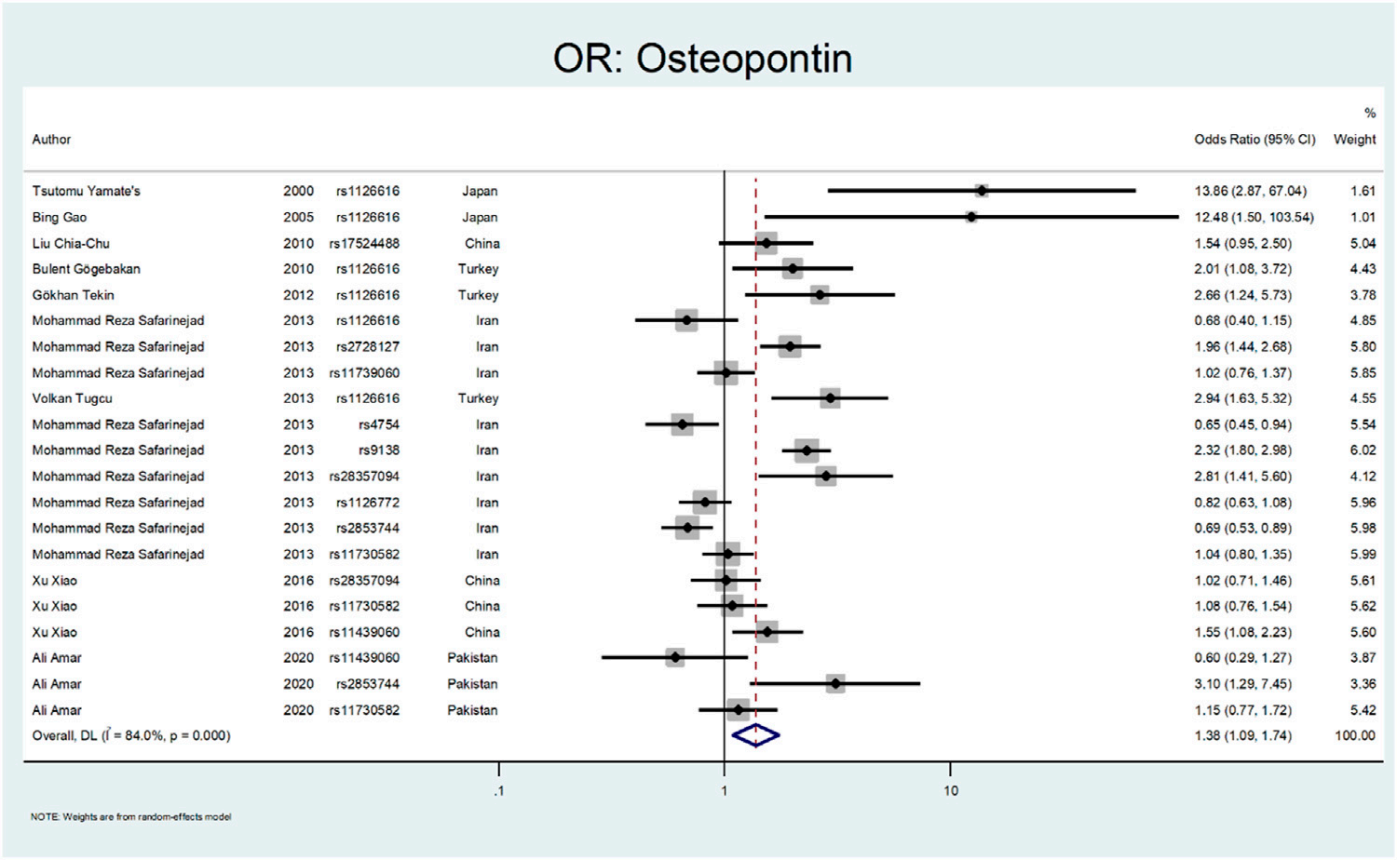
Forest plot: OR for Osteopontin.

**FIGURE 11:**
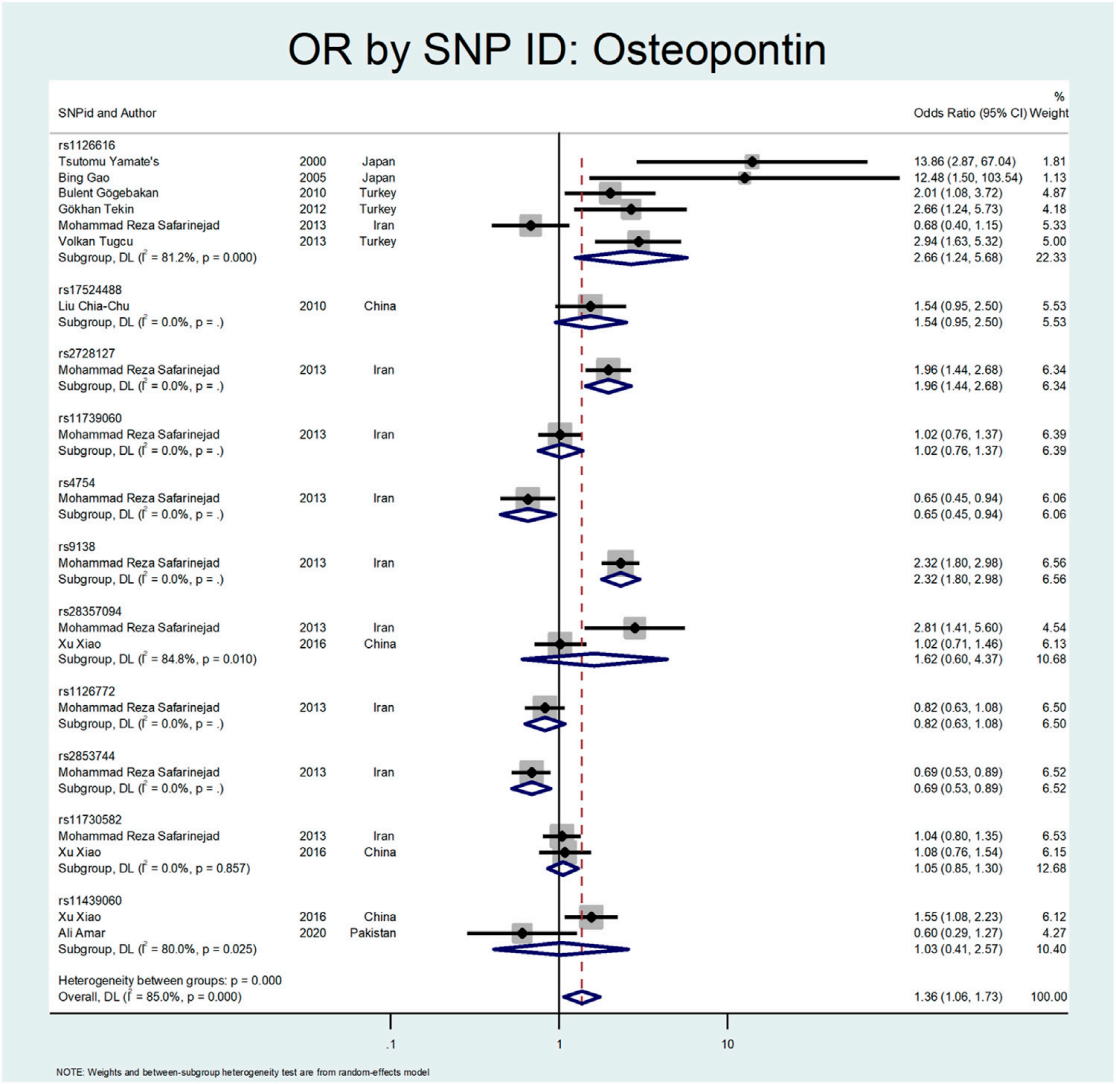
Subgroup analysis for Osteopontin, by SNP subgroup.

The results related to OR for Osteopontin (OPN) are shown in Figure 10 and Figure 11. This gene also showed a significant association (OR = 1.38; 95%CI:1.09–1.74). All studies on this gene were conducted in Asian countries, and subgroup analysis by continent was impossible. A significant odds ratio was found for the subgroup of rs1126616 (OR = 2.66; 95% CI:1.24–5.68), rs2728127 (OR = 1.96; 95%CI:1.44–2.68), and rs9138 (OR = 2.32; 95% CI:1.80–2.98). Furthermore, we found significant values of OR for rs2853744 (OR = 0.69; 95% CI:0.53–0.89), and rs4754 (OR = 0.65; 95% CI:0.45–0.94) as protective polymorphisms against nephrolithiasis. The estimated OR in other SNP subgroups of this gene was not statistically significant. A weak and insignificant association was observed for rs11439060 (OR = 1.02; 95% CI:0.76–1.37), and rs11730582 (OR = 1.05; 95% CI:0.85–1.30).

**FIGURE 12:**
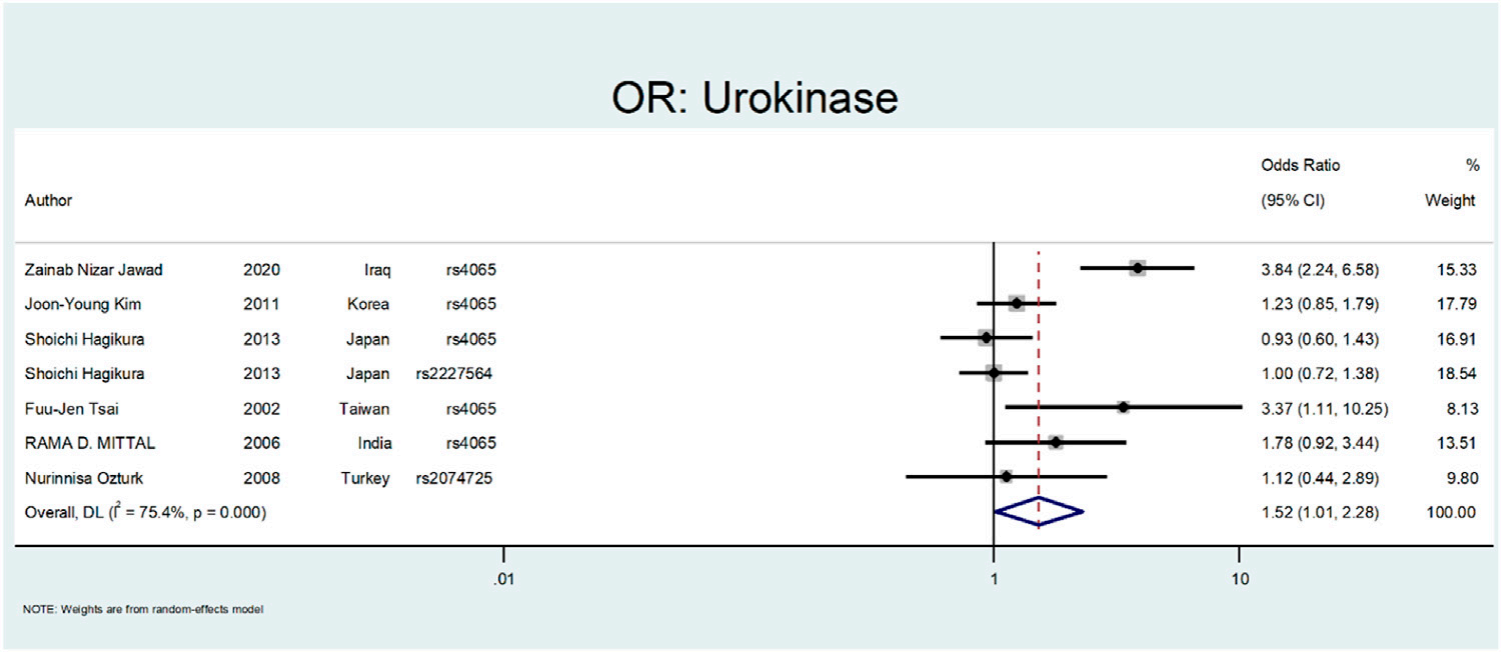
Forest plot: OR for Urokinase.

**FIGURE 13:**
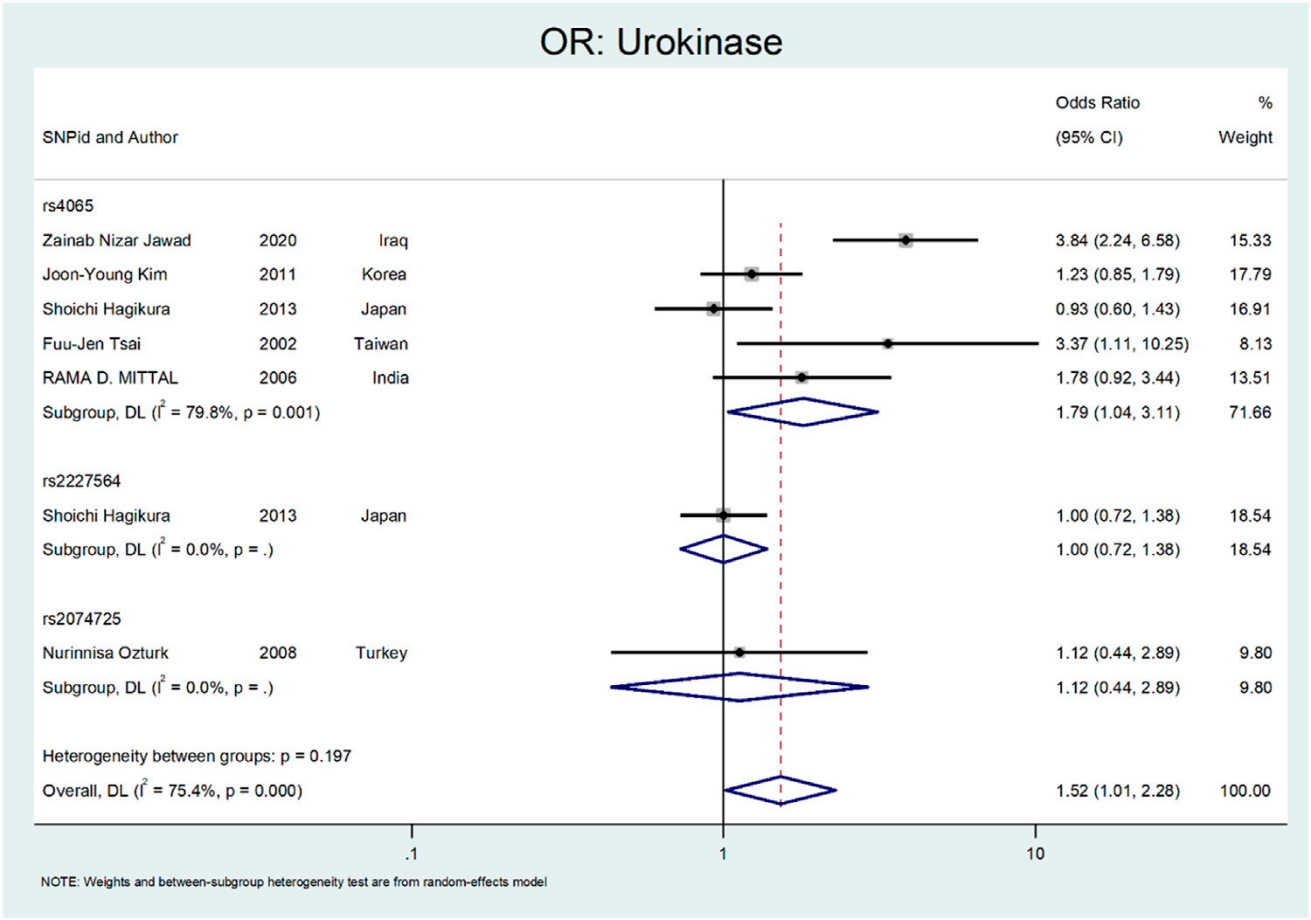
Forest plot: ORsubgroup analysis for Urokinase, by SNP subgroup.

Finally, we performed the meta-analysis for Urokinase (Figure 12 and Figure 13). The odds ratio was statistically significant (OR = 1.52; 95% CI: 1.02–2.28). A significant association was also found in SNP subgroup of rs4065 (OR = 1.79; 95% CI: 1.04–3.11), but the values in other SNP subgroups were not statistically significant (rs2227564, OR = 1.95 CI: 0.72–1.38 and rs2074725, OR = 1.12; 95% CI: 0.44–2.89).

**FIGURE 14:**
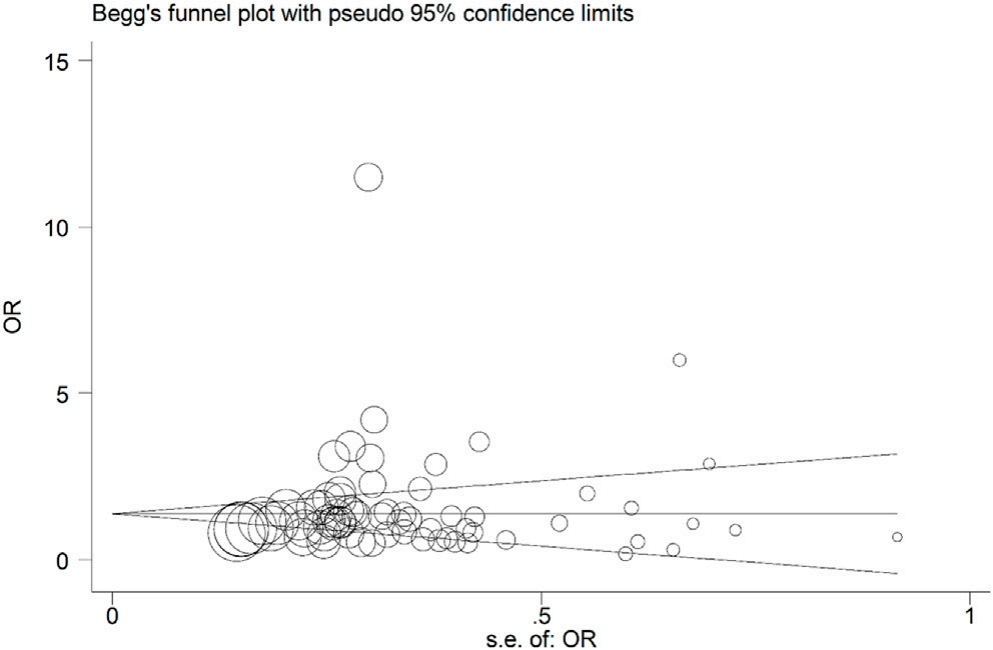
Funnel, Eagers’, & Beggs’ plots for assessment of publication bias.

**FIGURE 15:**
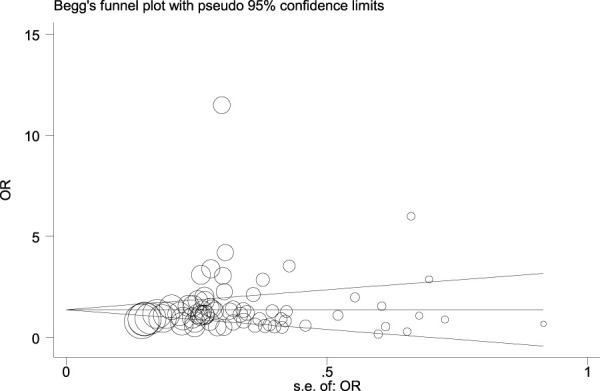
Funnel, Eagers’, & Beggs’ plots for assessment of publication bias.

**FIGURE 16:**
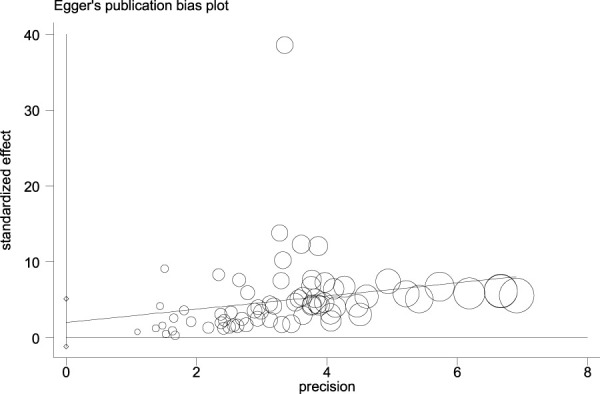
Funnel, Eagers’, & Beggs’ plots for assessment of publication bias.

Figure 14, Figure 15, and Figure 16 (funnel, eager’s, and Begg’s plots) show an ignorable publication bias in this study.

Table 1 shows the results of OR related to the effect of a gene and its SNPs in the Caucasian sub-population. This ethnicity was found in Turkey, Canada, and Italy. Two studies in Italy and Canada showed a protective OR for CASR with SNP subgroups rs1801726 (OR = 0.49; 95%; CI:0.17–1.42), and (OR = 0.95; 95% CI: 0.42–2.13), while a former study in Italy identified this SNP as a risk factor (OR = 1.32; 95%; CI:0.35–5.00). The other evaluated SNP in three different studies was rs1801725 (OR = 1.23; 95% CI:0.71–2.12, OR = 1.04; 95% CI: 0.68–1.58, and OR = 1.64; 95% CI: 1.09–2.46). The estimated value was significant only in a study by Hamilton et al. (OR:1.64; 95%; CI:1.09–2.46). In addition, studies on rs1042636 in Canada showed insignificant values of OR = 1.45; 95%; CI:0.78–2.69 and OR = 1.60; 95%; CI: 0.53–4.83, while this value was strongly significant in a study in Italy OR = 5.20; 95% CI: 1.71–15.79.

**TABLE 1:** Studies on Caucasian population.

First author	Gene	SNP subgroup	Year	Country	OR (95%CI)
Otzurk	Urokinase	rs2074725	2008	Turkey	1.12 (0.44–2.89)
Bulent Gögebakan	Osteopontin	rs1126616	2010	Turkey	2.01 (1.08–3.72)^a^
A. Scillitani	CASR	rs1042636	2007	Canada	1.45 (0.78–2.69)
D. C. Hamilton	CASR	rs1042636	2009	Canada	1.60 (0.53–4.83)
G. Vezzoli	CASR	rs1042636	2007	Italy	5.20 (1.71–15.79)^a^
S. Corbetta	CASR	rs1801725	2006	Italy	1.23 (0.71–2.12)
D. C. Hamilton	CASR	rs1801725	2009	Canada	1.64 (1.09–2.46)^a^
A. Scillitani	CASR	rs1801725	2007	Canada	1.04 (0.68–1.58)
S. Corbetta	CASR	rs1801726	2006	Italy	0.49 (0.17–1.42)
G. Vezzoli	CASR	rs1042626	2002	Italy	1.32 (0.35–5.00)
A. Scillitani	CASR	rs1801726	2007	Canada	0.95 (0.42–2.13)

a statistically significant.

## Discussion

Urinary stone disease is a complex phenomenon with a multifactorial etiology, with varying incidence in different continents and climates (5–20% in Asia, 5–9% in Europe, and 7–13% in North America) (; Sorokin et al., 2017). The most common urinary stones are calcium oxalate, calcium phosphate, struvite, uric acid, and cystine (Zhang et al., 2021). Dietary habits, occupation, metabolic disorders, urinary tract anomalies, climate, and genetic factors are proposed to contribute to stone formation (Mehrsai et al., 2019; Wood et al., 2019; Ebrahimi et al., 2021; Fakhr Yasseri et al., 2021). Many efforts have been made to elucidate the role of genetic factors, especially single-nucleotide polymorphism (SNPs), in renal stone disease. However, the multifactorial nature of stone formation impedes considering the SNPs alone. Also, the results of studies are contradictory regarding the role of specific SNPs in renal stone formation (Monico and Milliner, 2012; Liu et al., 2014; Palsson et al., 2019; Vladimirovna et al., 2020). Our systematic review and meta-analysis reviewed the literature in the last 20 years to evaluate the role of most cited SNPs in stone formation in different continents to gather relevant data for clinicians regarding the role of genetics in stone formation.

The most investigated gene in the literature related to the renal stone disease is the *vitamin D receptor gene (VDR)*. V*DR* gene has an essential role in calcium metabolism and is located in human chromosome 12q13–12q14 (Taymans et al., 1999; Gunes et al., 2006). There are four main genetic variations in the VDR gene, including rs2228570 (FokI), rs731236 (TaqI), rs7975232 (ApaI), and rs1544410 (BsmI) (Mossetti et al., 2003; Seo et al., 2010; Yang et al., 2019a; Amar et al., 2020a). From 1998 to 2021, seventy-two articles investigating the role of different SNPs in the *VDR* gene were included in the meta-analysis (Ruggiero et al., 1998; JACKMAN et al., 1999; Chen et al., 2001; Nishijima et al., 2002; Mossetti et al., 2003; Shaogang et al., 2003; Özkaya et al., 2003; Mossetti et al., 2004; Relan et al., 2004; Rendina et al., 2004; Gunes et al., 2006; Franco et al., 2007; Liu et al., 2007; Moyano et al., 2007; Seyhan et al., 2007; Mittal et al., 2010; Seo et al., 2010; Aji et al., 2012; Basiri et al., 2012; Wang et al., 2012; Aykan et al., 2014; Guha et al., 2015; Aykan et al., 2016; Cakir et al., 2016; Goknar et al., 2016; Aghamir et al., 2017; Rendina et al., 2017; Subasi et al., 2017; Wang et al., 2017; Ergon et al., 2018; Amar et al., 2019; Yang et al., 2019a; Yang et al., 2019b; Huang et al., 2019; Li et al., 2019; Jawad and Awad, 2020). The overall estimate of OR for each SNP in forest plots. The pooled estimate of ORs for *VDR* was around 1.20 (95%CI:1.06–1.36), showing a significant difference between the two study groups (patients with nephrolithiasis and healthy controls).

Among studies that had significant differences between case and control groups, Nishijima et al. evaluated the role of TaqI and ApaI in the pathogenesis of renal stones. They concluded that the incidence of TaqI Tt and tt genotypes was significantly higher in patients with nephrolithiasis with a 5.2 fold increase risk of stone formation. However, the ApaI was not significantly different between healthy and nephrolithiasis patients (Nishijima et al., 2002). Ozkaya et al. found that the ApaI AA genotype was significantly higher in nephrolithiasis patients (OR:2.27, CI = 1.21–3.42) (Özkaya et al., 2003). In two different studies by Redina et al. and Relan et al., the only genotype related to nephrolithiasis was rs1544410 (BsmI) with ORs 2.03 and 1.81, respectively (Relan et al., 2004; Rendina et al., 2004). Also, Relan et al. concluded that rs 2228570 (FokI) was associated with nephrolithiasis (OR:3.06; CI = 1.7–5.53) (Relan et al., 2004). In a study by Bid et al., the rs 2228570 (FokI) was associated with renal stone disease (OR:3.11; CI = 1.88–5.17) (Bid et al., 2005). Mittal et al. also found the rs 2228570 (FokI) relation with urolithiasis (OR:3.41; CI = 1.98–5.87) (Mittal et al., 2010). The rs731236 (TaqI) was a significant polymorphism in the *VDR* gene in a study by Wang et al., with OR of 11.5 (CI = 6.41–20.6) (Wang et al., 2012).

In Figure 3, Figure 4, sub-group analysis of this gene showed a significant association in Asian patients with an overall OR of 1.24 (95%; CI:1.08–1.42). However, the estimated OR in European and North American studies subgroups was insignificant (OR = 0.99; 95% CI: 0.75–1.32, and OR = 1.56; 95% CI: 0.48–5.10). Among SNP subgroups, the overall estimate for OR of rs2228570 showed a significant relationship (OR = 1.28; 95% CI:1.01–1.62). In a study by Imani et al., they investigated the role of *VDR* gene polymorphism regarding the increased risk of nephrolithiasis in a systematic review. Their results did not confirm the association of FokI, TaqI, BsmI, and ApaI with nephrolithiasis. However, ApaI and TaqI SNPs were related to urolithiasis in East-Asian and Caucasians populations. Our results revealed a significant association in the overall pooled meta-analysis (OR:1.20; 95% CI: 1.06–1.36). In subgroup analysis, rs2228570 (FokI) showed a significant relationship (OR = 1.28; 95% CI:1.01–1.62) with urolithiasis (Imani et al., 2020).

The *CASR* gene variation was the following gene that was explored. The *CASR* is a dimeric membrane protein belonging to the G-protein. It is mainly present in the parathyroid glands and thick ascending limb of Henle tubules and preserves calcium homeostasis (Brown et al., 1993). A total number of twenty-two studies was included in our study (Vezzoli et al., 2002; Corbetta et al., 2006; Scillitani et al., 2007; Vezzoli et al., 2007; Hamilton et al., 2009; Shakhssalim et al., 2010; Chou et al., 2011; Kim et al., 2011; Han et al., 2013; Vezzoli et al., 2013; Guha et al., 2015; Vezzoli et al., 2015; Ding et al., 2017; Li et al., 2018; Zhou et al., 2018; Chen et al., 2019; Litvinova et al., 2021). A study by Corbetta et al. evaluated 94 patients with primary hyperparathyroidism regarding *CASR* gene variants. They concluded that the R990G polymorphism was related to the hyperparathyroidism parameters. This variant R990G (rs1042636) was associated with recurrent urolithiasis (OR:3.76; CI = 1.39–10.22) (Corbetta et al., 2006). In a similar study by Vezzoli et al., on 124 hypercalciuric patients without a history of urolithiasis, they found the R990G polymorphism was related to the hypercalciuric state compared to the healthy normocalciuric patients (OR:5.2; CI = 1.71–15.79) (Vezzoli et al., 2007).

Shakhssalim et al. evaluated *CASR* polymorphism in ninety-nine patients with recurrent urinary stones compared to 107 healthy control. They found that the 990G (rs1042636) and 986S (rs1801725) were associated with recurrent calcium urolithiasis (ORs:7.24 and 2.62, respectively) (Shakhssalim et al., 2010). In two different studies by Vezzoli et al., the rs1501899 (OR:2.07; CI = 1.37–3.13), rs6776158 (OR:3.17, CI: 2.14–4.7), and rs1042636 (OR:1.88; CI:1.17–3.04) were related to the stone formation (Vezzoli et al., 2013; Vezzoli et al., 2015). A study by Guha et al. revealed that rs1042636 (OR:2.46; CI: 1.64–3.69) and rs1801725 (OR:3.09; CI: 1.97–4.85) of the CASR gene were meaningfully related to urolithiasis (Guha et al., 2015). In a study by Ding et al., the rs1042636 (OR:1.52; CI: 1.09–2.12) was recognized as a risk factor for stone formation (Ding et al., 2017). Li et al. confirmed the role of *CASR* rs7652589 polymorphism in the development of stone disease in the Chinese population (Li et al., 2018).

In our meta-analysis, the overall estimate of OR for the *CASR* gene showed a significant association with stone formation (OR = 1.24; 95% CI: 1.01–1.52). In the subgroup analysis by continents, the OR was statistically significant only in European and North American patients (OR = 1.56; 95% CI: 1.10–2.22, OR = 1.31; 95% CI: 1.02–1.67, respectively). Regarding SNP subgroups, the rs1801725 and rs1501899 served as a risk factor for urolithiasis with OR values of 1.44 (95% CI: 1.06–1.97) 1.65 (95% CI: 1.08–2.51), respectively.

The third evaluated gene was the Klotho gene. This gene is a type-1 transmembrane protein with glucuronidase activity that has a proposed role in renal calcium and phosphate homeostasis (Telci et al., 2011). In five studies, ten Klotho gene variation was evaluated (Telci et al., 2011; Xu et al., 2013; Gürel et al., 2016). Telci et al. evaluated three SNPs in the Klotho gene and found the protective effect of rs3752472 on urolithiasis (Telci et al., 2011). In a study by Xu et al., they assessed the relationship between Klotho gene polymorphism and nephrolithiasis. They concluded that the rs3752472 polymorphism of the Klotho gene has a protective effect on the stone formation (OR:0.66; CI = 0.44–0.99) (Xu et al., 2013). Gurel et al. investigated the Klotho gene polymorphism and found that the rs1207568 variation has protective effects on the stone formation (OR:0.41; CI: 023–0.73) (Gürel et al., 2016).

Our analysis shows that the Klotho has a protective effect on the outcome of interest (OR = OR = 0.75; 95% CI: 0.57–0.99). All studies on this gene were conducted in Asian countries, so we did not perform subgroup analysis by continent. According to Figure 9, a significant protective odds ratio was only observed in rs3752472 (OR = 0.58; 95% CI: 0.39–0.86).

The following gene that was evaluated is *Osteopontin* (Liu et al., 2010). Osteopontin is a multifunctional glycosylated phosphoprotein. Many human and animal studies have proposed this gene polymorphism as a factor in renal stone formation (Katsuma et al., 2002). Yamate et al. investigated the role of *Osteopontin* in hereditary nephrolithiasis cases. They found the rs1126616 was significantly detected in renal stone cases (OR:13.86; 95% CI: 2.87–67.04) (Yamate et al., 2000). Gao et al. evaluated the role of osteopontin polymorphism on 76 patients with urolithiasis. They found a significant difference in rs1126616 variation in nephrolithiasis patients compared to the healthy patients (OR:12.48; 95% CI: 1.5–103.54) (Gao et al., 2005). Gogebakan et al. performed another study on osteopontin polymorphism in renal stone patients. Their results revealed the role of rs1126616 polymorphism in developing nephrolithiasis (OR:2.01; 95%; CI = 1.08–2.72) (Gögebakan et al., 2010).

In a study by Tekin et al., they evaluated 65 pediatric patients with nephrolithiasis regarding *OPN* gene polymorphism. The rs1126616 was significantly discovered in kidney stone cases (OR:2.66; 95% CI = 1.24–5.73) (Tekin et al., 2012). Safarinejad et al. evaluated SNPs in the osteopontin gene on 342 patients with calcium oxalate stones. Their results revealed that rs28357094 TT genotype (OR:2.52; 95% CI = 1.74–3.79; *p* = 0.021), rs2728127 GG genotype (OR: 2.64; 95% CI1.42–4.81; *p* = 0.002), and rs2853744 GG genotype (OR 1.68; 95% CI: 1.22–3.87; *p* = 0.003) had a strong relation with nephrolithiasis. However, the rs9138 AA genotype was protective regarding stone formation (OR 0.62, 95% CI 0.47–0.81; *p* = 0.004) (Safarinejad et al., 2013). Tugcu et al. evaluated *OPN* gene polymorphism in 127 urolithiasis patients and found the rs1126616 polymorphism had a significant difference between case and control groups (OR:2.94; 95%CI = 1.63–5.32) (Tugcu et al., 2013). Amar et al. found three SNPs in the OPN gene related to stone formation. The rs2853744:G > T, rs11730582:T > C and rs11439060:delG > G, were significantly linked with stone formation (OR = 3.14; *p* = 0.006, OR = 1.78; *p* = 0.006 and OR = 1.60; *p* = 0.012, respectively) (Amar et al., 2020b).

In a study by Xiao et al., they evaluated the role of *OPN* polymorphism in renal stone formation. In their study, three SNPs, including rs28357094, rs11439060, and rs11730582, were genotyped in 230 renal stone patients. The only SNP that related to nephrolithiasis was rs11439060 compared with the controls (OR:1.55; 95% confidence interval (CI) = 1.08–2.22) (Xiao et al., 2016).

Our results related to OR for Osteopontin are shown in Figure 10, Figure 11. This gene also showed a significant effect overall (OR = 1.38; 95%CI:1.09–1.74). The rs1126616 (OR = 2.66; 95%CI:1.24–5.68), rs2728127 (OR = 1.96; 95%CI:1.44–2.68), and rs9138 (OR = 2.32; 95%CI:1.80–2.98) were associated with risk of nephrolithiasis. The estimated OR in other SNP subgroups of this gene was not statistically significant.

The last gene that was evaluated is *Urokinase*. The *Urokinase* is a multifunctional protein produced in the kidney. The proposed mechanism in nephrolithiasis inhibitory affects calcium oxalate crystallization (Wagner et al., 1996; Morovvati et al., 2016). In a study by Tsai et al., they investigated the role of Urokinase gene polymorphism in 153 patients with recurrent calcium stones. Their results revealed that the Urokinase gene 3′-UTR C/T polymorphism is a risk factor for stone formation (OR:3.088; 95% CI = 1.06–8.99, *p* < 0.05) (Tsai et al., 2002). In a similar study by Mittal et al., they did not find a relation between urokinase gene 3′-UTR C/T (rs4065) polymorphism and risk of stone formation (OR:1.78; 95% confidence interval = 0.92–3.44) (Mittal et al., 2006). Ozturk et al. evaluated the role of 3′-UTR C/T polymorphism in urolithiasis. They did not find any relation between this polymorphism and calcium stone formation (OR:1.12; CI = 044–2.89). However, this polymorphism was more frequent in children and recurrent urinary stone cases (Ozturk et al., 2008). Kim et al. evaluated the role of 3′-UTR C > T (rs4065) polymorphism in renal calcium stone in the Korean population. There are no significant differences between patients and the control group regarding this polymorphism (OR:1.23; CI = 085–1.79) (Kim et al., 2011). In a study by Hagikure et al., they evaluated the role of P141L (rs2227564) and 3′-UTR C > T (rs4065) in 232 patients with calcium stones in the Japanese population. Their results revealed no clear relationship between these two polymorphisms and the risk of urolithiasis (OR:1.00; CI = 072–1.38, and OR:093; CI = 0.6–1.43, respectively) (Hagikura et al., 2013). Jawad et al. evaluated the role of 3′-UTR C > T (rs4065) polymorphism in renal calcium stone in the Iraqi population. There was a significant relationship between this polymorphism and the risk of stone formation (OR:3.84; CI = 2.24–6.58) (Kim et al., 2011).

Figures 12, 13 depict the meta-analysis for *Urokinase* gene polymorphism. The overall odds ratio was statistically insignificant (OR = 1.52; 95%CI:1.02–2.28). A significant association was also found in SNP subgroup of rs4065 (OR = 1.79; 95% CI:1.04–3.11), but the values in other SNP subgroups were not statistically significant (rs2227564 with OR = 1; 95%CI:0.72–1.38) and (rs2074725 with OR = 1.12; 95%CI:0.44–2.89).

The overall publication bias of our study (Figure 14, Figure 15, Figure 16) was acceptable. One of the distinguished structures of our research is that we evaluated the role of Caucasian ethnicity in stone formation separately. The main reason for this categorization was that many studies showed that Caucasian ethnicity significantly influenced polymorphism (Vezzoli et al., 2002; Corbetta et al., 2006; Scillitani et al., 2007; Vezzoli et al., 2007; Ozturk et al., 2008; Hamilton et al., 2009; Gögebakan et al., 2010). The three countries included in the study with Caucasian ethnicity were; Italy, Turkey, and Canada. Some studies mentioned that the Caucasian subpopulation is linked with an increased risk of urolithiasis, and this subpopulation has a worldwide distribution (Riley et al., 2018).

Vezzoli et al. found that *CASR* gene polymorphism (rs1042636) was associated with nephrolithiasis risk in this ethnicity (OR:5.20; CI = 1.71–15.79). A study by Hamilton et al. found a significant relation between *CASR* gene polymorphism (rs1801725) and nephrolithiasis (OR:1.64; CI = 1.09–2.46) in the Caucasian population. Gögebakan et al. found an association between osteopontin gene polymorphism (rs1126616) and nephrolithiasis in the Caucasian population (OR:2.01, CI = 1.08–3.72) (Gögebakan et al., 2010).

Our study has some limitations. First, some of the studies were in Asia, but we do not have any data regarding the patients’ ethnicity. Multi-ethnicity may be considered as a confounding factor. The second one is restraining the search to only English-language articles. Third, most studies on *Osteopontin, Klotho,* and *Urokinase* gene polymorphisms were performed in Asia, so we recommend more studies regarding these genes in other continents, including Europe and North America.

## Conclusion

The multifactorial nature of the stone formation, emphasizing the role of environmental factors, might explain contradictory results in the literature. While polymorphisms in *VDR, CASR,* Osteopontin, and Urokinase genes were associated with urinary stone formation, the Klotho gene showed a protective effect.

## Data Availability

The raw data supporting the conclusions of this article will be made available by the authors, without undue reservation.
